# Biological and chemical compositions of atmospheric particulate matter during hazardous haze days in Beijing

**DOI:** 10.1007/s11356-018-3355-6

**Published:** 2018-10-12

**Authors:** Zhendong Guo, Zhongyi Wang, Lu’an Qian, Zongzheng Zhao, Chunmao Zhang, Yingying Fu, Jiaming Li, Cheng Zhang, Bing Lu, Jun Qian

**Affiliations:** 1 Key Laboratory of Jilin Province for Zoonosis Prevention and Control, Changchun Veterinary Research Institute, Chinese Academy of Agriculture Sciences, Liu Ying Road 666, Changchun, 130122 Jilin China; 2 Academy of Military Medical Sciences, Beijing, China; 3 The Experimental High School Attached to Beijing Normal University, Beijing, 100032 China

**Keywords:** Atmospheric particulate matter, Biological and chemical compositions, Hazardous haze days, Health impact, Air quality index, Beijing

## Abstract

Particulate matter (PM), a major air pollutant in Beijing in recent years, poses a formidable public health threat. Even through many studies have documented the chemical and biological characteristics of PM, less is known about these characteristics on hazardous haze days (Air Quality Index, AQI 301–500) and the difference with sunny or unhealthy haze day (AQI 151–200) characteristics. Herein, studies were performed during a red alert air pollution event (continuous hazardous haze days) and the first few days following the event (sunny days first and then unhealthy haze days) in Beijing from December 19 to 25, 2016. A laser particle counter and an ANDERSEM-6 sampler were used to study the concentration and size distributions dynamics of the PM and the culturable airborne bacteria and fungi, respectively. PM_2.5_ was sampled by a high-volume air sampler and the chemical compositions, bacterial and fungal community structures, and endotoxin levels were analyzed. The results showed that the PM concentrations on the hazardous haze days and unhealthy haze days were 10.7 and 8.0 times higher, respectively, than those on the sunny days. The chemical composition of PM_2.5_ was highly correlated with the AQI. The concentration and percentage of water-soluble inorganic ions (WSII), which dominated the PM_2.5_ constituents, as well as the levels of endotoxin were higher on hazardous haze days than on unhealthy haze days and sunny days. Interestingly, the abundances of bacteria and fungi demonstrated the following order: unhealthy haze days> sunny days> hazardous haze days. Most culturable bacteria and fungi were distributed in particles with aerodynamic diameters of 2.1–4.7 μm. Redundancy analysis found total organic carbon explained 30.0% and 27.1% of total variations in bacterial composition and fungal composition at the genera level, respectively. Our results facilitate a better understanding of the biological and chemical composition dynamics of PM in Beijing.

**Electronic supplementary material:**

The online version of this article (10.1007/s11356-018-3355-6) contains supplementary material, which is available to authorized users.

## Introduction

Particulate matter (PM) is a complex mixture of particles with different bioaerosol and chemical compositions in different sampling locations and seasons and this componential variety may contribute to PM toxicity (Seagrave et al. 2006; Janssen et al. 2011). Many human diseases involving the respiratory and cardiovascular systems have been found to be related to PM exposure (Langrish et al. 2012). Microbial or chemical components attached to PM could potentially affect human health by targeting different body regions, such as mucosa, the skin, the digestive tract, and the respiratory tract (Stahlhofen et al. 1980; Haas et al. 2013). Previous studies have shown that prolonged exposure to PM_2.5_ may increase the risk of developing lung cancers (Abba et al. 2012).

Air pollution in the northern cities of China has been one of the most significant problems in the process of social and economic development. Beijing is the largest city in the North China Plain, which has a northeaster winter monsoon from the Mongolian Plateau. The annual average concentration of PM_2.5_, defined as minute particles less than or equal to 2.5 μm in aerodynamic diameter, is 70–100 μg/m^3^ in Beijing, which is 2–3 times higher than the National Air Quality Standard (Wang et al. 2015).

During winter days in the northern cities of China, heating consumes a high amount of fuel, which might contribute to high PM_2.5_ values and low atmospheric visibility. It is well known that PM_2.5_ can generate toxic effects by targeting respiratory surfaces and dissolving into blood (Cao et al. 2014). The microbial or chemical components attached to PM_2.5_ on haze days may differ from those on sunny days due to environmental changes that affect the survival of microorganisms and the different sources generating the PM. Although some studies have focused on microbial and chemical analyses of PM_2.5_ during haze days (Kunzli et al. 2006; Lippmann and Chen 2009), data from hazardous haze days are limited, and it is still unclear how the biological and chemical contents change during the transition between sunny days and haze days and whether this change could potentially affect human health.

From December 16 to December 21 in 2016, there was a heavy air pollution situation in North China, and the Beijing government issued the third red alert in history. From December 22 to December 23, the red alert was lifted, and the air quality became excellent. However, from December 24 to December 25, the air quality became slightly polluted, a condition that lasted for 2 days. During this heavy air pollution period, although the peak value of AQI did not exceed 500 and was lower than 508 (the peak value in 2015), the haze period lasted for more days than the two red alerts in 2015. From the perspective of scientific research, this event provided a good opportunity to explore whether the heavily polluted air conditions could influence the survival of microorganisms and how the microbial or chemical components attached to the PM_2.5_ changed during the transition between sunny days and haze days.

Here, we studied the concentration and size distribution dynamics of atmospheric PM and culturable airborne bacteria and fungi from December 19 to 25, 2016. Simultaneously, the chemical compositions, bacterial and fungal community structures, and endotoxin levels of PM_2.5_ samples were analyzed. The results of this study may offer insight into the biological and chemical composition dynamics of PM on hazardous haze days, unhealthy haze days and sunny days.

## Materials and methods

### Particulate matter collection

**Fig. 1:**
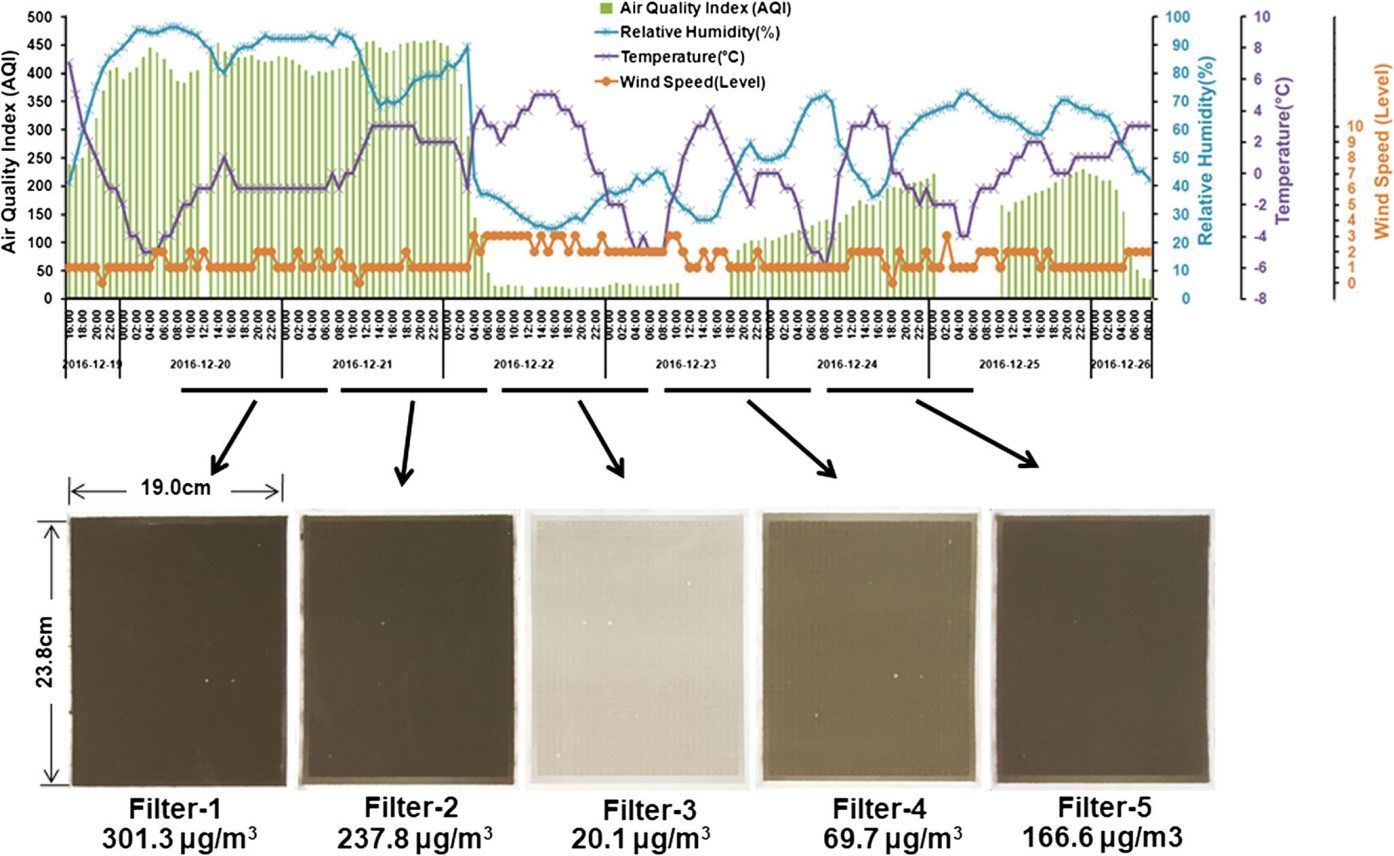
Meteorological conditions and PM concentrations during the study period. Meteorological data including the Air Quality Index (AQI), temperature, relative humidity, and wind speed were collected from the China Weather website (http://www.weather.com.cn/weather/101010100.shtml). The PM_2.5_ samples were collected on a quartz fiber filter (19.0 cm × 23.8 cm) using a high-volume sampler at a flow rate of 1000 L/min. From December 20 to December 25, five air filters were used for five 23-h sample collections. All sampled filters were then preserved in the dark at − 20 °C until the chemical and biological analyses. The filter weights were measured before and after the sampling procedure. The PM concentrations for the five sampling days are also shown in the lower part of this figure

The PM_2.5_ sampling was performed in an open-space area without major pollution sources nearby, ∼ 2 m above the ground on the campus of the Beijing Institute of Technology (39° 57′ 51.0″ N; 116° 19′ 38.5″ E). The PM_2.5_ samples were collected on 20.32 × 25.4-cm^2^ Tissuquartz filters (PALL, NY, U.S.) using a high-volume air sampler equipped with a PM_2.5_ fractionating inlet (Beijing Huarui Hean Technology Co., Ltd., China) at a flow rate of 1000 L/min. Five 23-h sample collections (8:00 AM–7:00 AM of the next day) were performed from December 20 to December 25 (Fig. 1). All the filters were sterilized by baking in a Muffle furnace at 500 °C for 48 h prior to sampling. The mass PM_2.5_ concentration was estimated by the net weight of the filter before and after the sampling divided by the flow-through volume. All sampled filters were then preserved in the dark at − 20 °C until the chemical and biological analyses.

### Meteorological conditions

Meteorological data including AQI, temperature, relative humidity, and wind speed were collected from the China Weather website (http://www.weather.com.cn/weather/101010100.shtml) and recorded per hour.

The AQI from 0 to 500 was divided into 6 categories, including hazardous (301–500), very unhealthy (201–300), unhealthy (151–200), unhealthy for sensitive groups (101–150), moderate (51–100), and good (0–50). In this study, days with AQI values below 100 were categorized as sunny days, while haze days had an AQI value greater than or equal to 100.

### Particulate matter number data monitoring

A laser particle counter (model 9306, TSI Inc., MN, USA) was used to monitor the number and size distribution of PM in the ambient air. The counter could measure up to six channels of simultaneous data, namely, 0.3–0.5 μm, 0.5–1 μm, 1–3 μm, 3–5 μm, 5–10 μm, and > 10 μm. Data were recorded every 5 mins. The sampling rate of the laser particle counter was 2.83 L/min, and the air sampling time was 10 s.

### Collection of culturable airborne bacteria and fungi

An international standard ANDERSEN-6 sampler (TE-10-800, Tisch Environmental, Inc., US) was used to sample the culturable airborne bacteria and fungi at a flow rate of 28.3 L/min, with Columbia blood agar (CM0331, Thermo Fisher Scientific Inc., US) and sand fort weak training bases (S9710, Beijing Solarbio Science & Technology Co., Ltd., China) as the sampling media, respectively. The Columbia blood agar base was composed of special peptone (23 g/L), starch (1 g/L), sodium chloride (5 g/L), agar (10 g/L), and 5% sterile defibrinated blood. The sand fort weak training bases were composed of peptone (10 g/L), glucose (40 g/L), and agar (20 g/L). The sampling time was 35 min. The sampler was sufficiently disinfected with 75% ethyl alcohol after each sampling. The ANDERSEN-6 sampler had six stages defined by the aerodynamic diameters of the airborne particles, namely, stage 6 (0.65–1.1 μm), stage 5 (1.1–2.1 μm), stage 4 (2.1–3.3 μm), stage 3 (3.3–4.7 μm), stage 2 (4.7–7.0 μm), and stage 1 (> 7.0 μm).

The airborne bacteria collection plates were cultured in 37 °C for 24–48 h, and the airborne fungi collection plates were cultured in 25 °C for 72 h; then, the bacteria and fungi colony numbers were counted on the sample dishes at each stage. The aerosol concentration (AC) of the culturable bacteria or fungi was calculated as: *AC=CN/(Q*_*s*_ *•* T_s_*)*, where *CN* is the colony number in the collection plates (CFU), *Q*_*s*_ is the sampler volumetric flow rate (L/min), and *T*_*s*_ is the sample collection duration (min).

### Chemical composition analysis

A quarter of each Tissuquartz filter sample and blank filter were cut into pieces, and the filter treating process was conducted as described in a previous study (Yang et al. 2016). Heavy metal ions (Hg, Pb, Cd, As and Cr) and water-soluble inorganic ions (NO_3_^−^, SO_4_^2−^, Cl^−^, NH_4_^+^, Mg^2+^, Na^+^, Ca^2+^, K^+^, et al.) were analyzed by ion chromatography (IC, Dionex 2100 for anions and Dionex 600 for cations, USA). The total organic carbon (TOC) concentrations were detected using a thermal-optical transmittance aerosol carbon analyzer (Sunset Laboratory Inc.)

### Biological composition analysis

For bacterial and fungal structure analysis, 1/8 of each Tissuquartz filter sample was used for DNA extraction by the MO-BIO PowerSoil DNA isolation kit (Carlsbad, CA, U.S.A.) according to the manufacturer’s protocols. Quantitative real-time polymerase chain reaction (Q-RT-PCR) was performed to quantify the relative abundance of bacteria and fungi among the five sampling filters. The primers were as follows: for bacterial *16sDNA*, 515F (5′- GTGCCAGCMGCCGCGGTAA-3′) and 806R (5′- GGACTACHVGGGTWTCTAAT-3′) were used; for fungal *ITS*, ITS1 (5’-TCCGTAGGTGAACCTGCGG-3′) and ITS1 (5’-GCTGCGTTCTTCATCGATGC-3′) were used. Q-RT-PCR assays were run on an Applied Biosystems® 7500 Real-Time PCR System (ThermoFisher SCIENTIFIC, U.S.A.).

For bacterial and fungal community structures, the V1–V3 regions of the bacterial 16S ribosomal DNA gene and the ITS region of the fungal rRNA operon were amplified by PCR (94 °C for 5 min, followed by 10 cycles of 94 °C for 30 s, 60 °C -55 °C for 45 s, and 72 °C for 90 s, 20 cycles of 94 °C for 30 s, 55 °C for 45 s and 72 °C for 90 s, and a final extension at 72 °C for 5 min). The primers were as follows: for bacteria, V1-9F (5′-CCTATCCCCTGTGTGCCTTGGCAGTCTCAGACGAGTTTGATCMTGGCTCAG-3′) and V3-541R (5′-CCATCTCATCCCTGCGTGTCTCCGACTCAG-barcode-ACWTTACCGCGGCTGCTGG-3′) were used, and for fungi, ITS-3F (5’-CCTATCCCCTGTGTGCCTTGGCAGTCTCAGCACATCGATGAAGAACGCAGC-3′) and ITS-4R (5’-CCATCTCATCCCTGCGTGTCTCCGACTCAG-barcode-GCTCCTCCGCTTATTGATATGC-3′) were used. The 20 μL PCR reaction mixture included 2 μL of 2.5 mM dNTPs, 4 μL of 5× FastPfu Buffer, 0.8 μL of each primer (5 μM), 0.4 μL of FastPfu Polymerase, and 10 ng of template DNA dissolved in 12 μL of deionized water (TransGen Inc., Beijing, China). The subsequent DNA purification, quantification, and pyrosequencing processes were the same as described in the previous study (Wei et al. 2016).

The endotoxins in the suspension used for chemical composition analysis were detected by the Limulus amebocyte lysate (LAL) (Associates of Cape Cod Inc., East Falmouth, MA). All the operations were conducted according to product instructions (Pyrotell-®T Multi-Test Kit).

### Statistical analysis

GraphPad Prism version 6.0 was used to perform statistical analyses, and a *p* value of less than 0.05 indicates a significant difference between groups. Pearson correlation analyses were used to assess the relationship of AQI with relative humidity, wind speed and chemical compositions. One-way analysis of variances (ANOVA) was used to analyze the difference of PM concentrations on haze days and sunny days, and the concentration difference of endotoxin and microbes was analyzed by independent sample *t* test analysis.

Relationship between airborne microbial composition and environmental factors (water-soluble inorganic ions, heavy metal ions, TOC and endotoxin level) was determined with redundancy analysis (RDA) which was implemented in the R software package. The total water-soluble inorganic ions concentration, the total heavy metal ions concentration, the TOC concentration, and the endotoxin concentration were introduced as explanatory variables. The relative contributions of the 21 bacterial genus-level or 10 fungal genus-level (average relative abundance > 1% in at least one sample) phylogenetic groups were used as response variables. Variance inflation factor analysis was used to analyze the collinearity of environmental factors, and the most colinear factor was deleted until no collinearity existed.

## Results

### Meteorological monitoring data and PM_2.5_ sampling

The study period ranged from December 19 to December 26 in 2016, including hazardous haze days (approximately 51 h, AQI > 400), sunny days (approximately 36 h, AQI < 50) and unhealthy haze days (approximately 48 h, 100 < AQI < 200). Paired pictures of hazardous haze days and sunny days are shown in Fig. S1. Meteorological data during this study including AQI, temperature, relative humidity and wind speed are shown in Fig. 1. There was no rainfall during this study period. A significant correlation between the AQI (*r* = 0.91) and the relative humidity was found. There was a moderate negative correlation between the wind speed and the AQI (*r* = − 0.56), indicating that higher humidity and lower wind speed might favor the accumulation of atmospheric pollutants.

A total of 5 PM_2.5_ samples were collected and named Filter-1 to Filter-5. As observed in Fig. 1, Filter-1 and Filter-2 were collected in hazardous haze days, Filter-3 was collected in sunny days and Filter-4 and Filter-5 were collected in unhealthy haze days. The PM concentrations estimated from the collected samples were 301.3, 237.8, 20.1, 69.7, 166.6 μg/m^3^ for Filter-1, Filter-2, Filter-3, Filter-4, and Filter-5, respectively.

### PM change dynamics during haze and sunny days

**Fig. 2:**
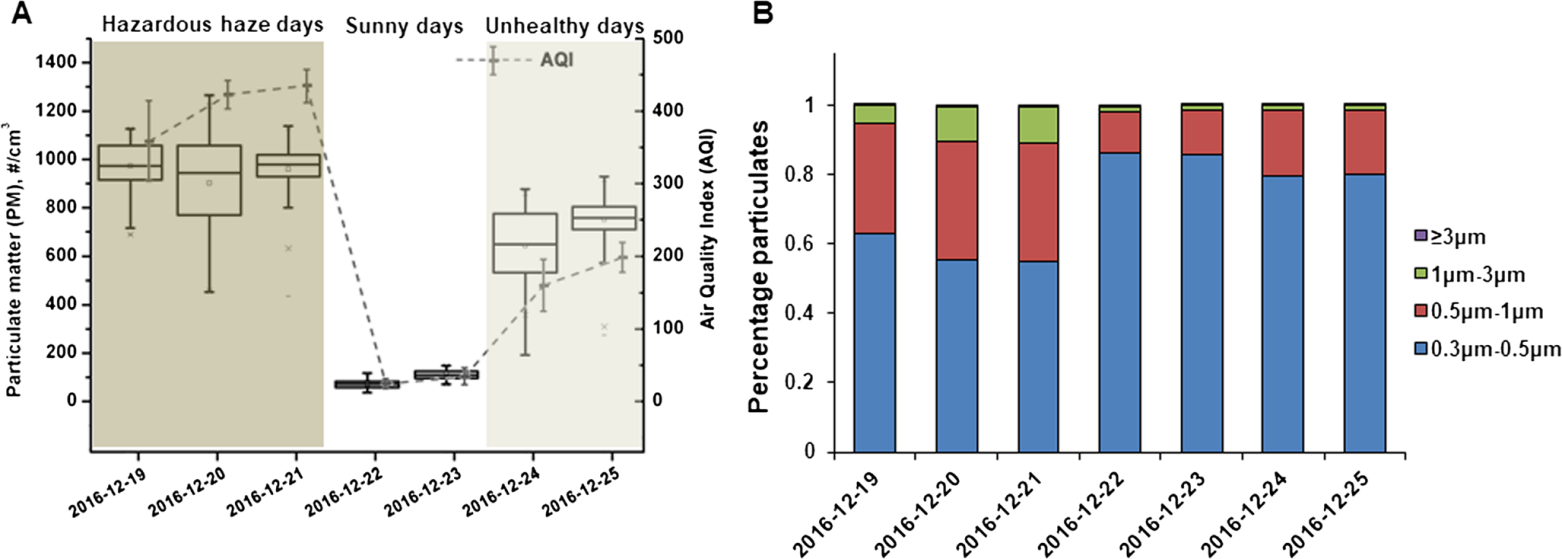
Boxplot of PM number (> 0.3 μm) during the study period (**a**) and the average size distribution maps of PM (**b**). Data from December 19 (19:00–24:00), December 20 (00:00–24:00) and December 21 (00:00–24:00) were used to represent the hazardous haze days. Data from December 22 (03:00–24:00) and December 23 (00:00–16:00) were used to represent the sunny days. Data from December 24 (00:00–24:00) and December 25 (00:00–24:00) were used to represent the unhealthy haze days

The PM concentration and size distributions during the study period were monitored by a laser particle counter (TSI, U.S.A.). The data were recorded every 5 min. As shown in Fig. 2a, the PM concentrations on the hazardous haze days (AVG 936.6 particles/cm^3^) and unhealthy days (AVG 697.7 particles/cm^3^) were significantly higher (10.7 and 8.0 times) than those on sunny days (AVG 87.5 particles/cm^3^). Figure 2b shows the particle size distributions on haze and sunny days. The number of particles smaller than 3 μm and 1 μm accounted for more than 99.5% and 89.0% of the total number of particles, respectively. The percentage of particles with aerodynamic diameters of 0.5 μm–1 μm and 1 μm–3 μm were significantly higher on hazardous haze days (AVG 34.1% and 9.7%) than on sunny days (AVG 12.2% and 1.4%). When the AQI deteriorated from sunny to unhealthy, the percentage of particles with aerodynamic diameters of 0.5 μm–1 μm increased (AVG 18.6%).

### Chemical composition analysis

**Fig. 3:**
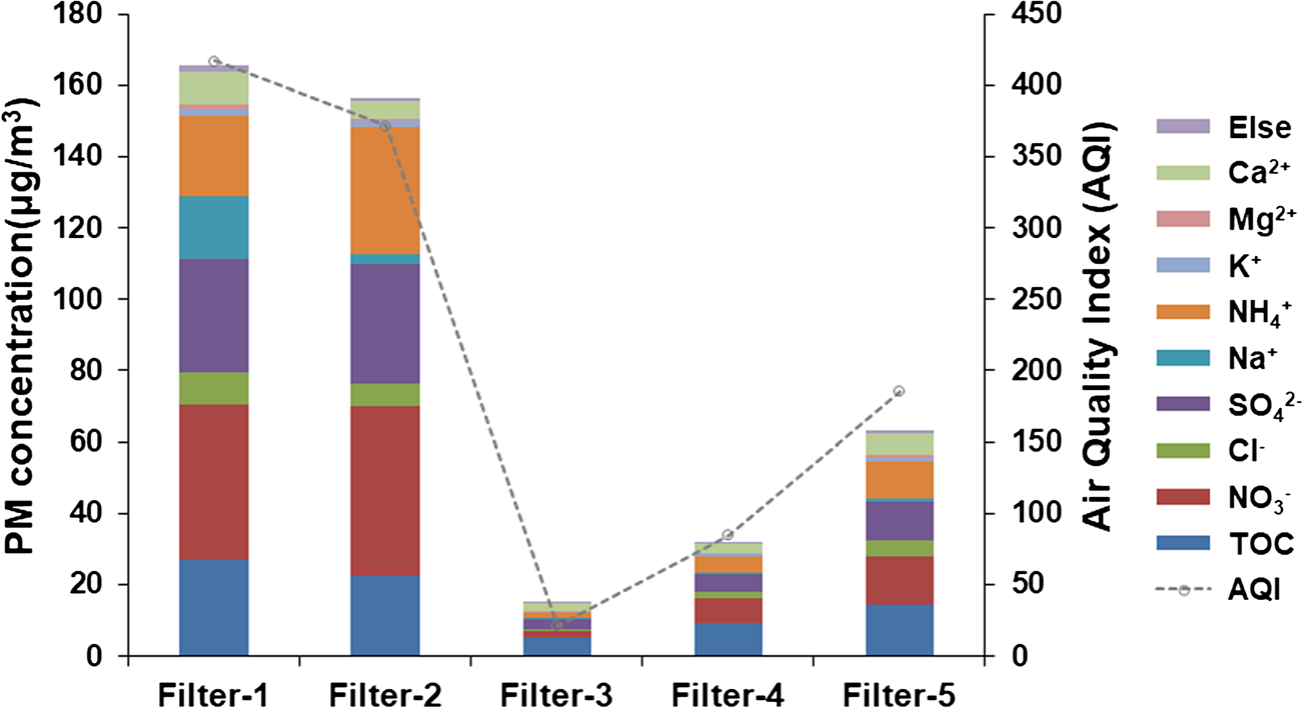
Mass concentration of PM and the PM chemical compositions during the sampling days. The dotted line represents the Air Quality Index (AQI) of all the sampling days from December 20 to December 25. The total organic carbon (TOC) and eight water-soluble inorganic ions, namely, Ca^2+^, Mg^2+^, K^+^, NH^4+^, Na^+^, SO_4_^2−^, Cl^−^, and NO_3_^−^ are shown. Details of other chemical compositions (described as Else), including other water-soluble inorganic ions (Zn^2+^, Cu^2+^, Fe^3+^, Al^3+^, and Ni^2+^) and heavy metal ions (Hg, Pb, Cd, As, and Cr) are shown in Table S1

The concentrations of water-soluble inorganic ions (WSII), heavy metal ions, and total organic carbon (TOC) during the study period were analyzed and are shown in Fig. 3 and Table S1. The levels of the PM_2.5_ and its chemical components during the study period had almost the same variation trend and were strongly correlated with the AQI.

Water-soluble inorganic ions (WSII) including NO_3_^−^, Cl^−^, SO_4_^2−^, NH_4_^+^, K^+^, Na^+^, Mg^2+^, and Ca^2+^ are important parts of atmospheric particles. The total concentrations of WSII on hazardous haze days, sunny days, and unhealthy haze days were 138.41, 9.78, and 48.64 μg/m^3^, respectively. The percentage of WSII in the PM_2.5_ on haze days was higher than that on sunny days. The percentage of WSII in the PM_2.5_ was 83.7% on hazardous haze days, 77.1% on unhealthy haze days, and 65.2% on sunny days. SO_4_^2−^, NH_4_^+^, and NO_3_^−^ dominated the WSII, accounting for 71.9 ± 9.7% of the WSII concentration. The concentrations of SO_4_^2−^, NH_4_^+^, and NO_3_^−^ were 16.8 and 5.9 times higher on hazardous haze days and unhealthy haze days, respectively, than those on sunny days.

Carbonaceous fractions were important components of the PM_2.5_ in the urban atmosphere. The TOC concentrations were higher on haze days than that on sunny days, with values of 26.9, 5.2 and 14.4 μg/m^3^ on hazardous haze days, sunny days and unhealthy haze days, respectively. However, the TOC percentage in the PM_2.5_ on haze days was lower than that on sunny days, with values of 16.3, 34.8 and 22.9 on hazardous haze days, sunny days, and unhealthy haze days, respectively.

The total concentrations of the measured heavy metal ions (Hg, Pb, Cd, As, and Cr) were 1.5, 0.23 and 0.85 μg/m^3^, accounting for 0.92%, 1.56%, and 1.35% of the PM_2.5_ mass on hazardous haze days, sunny days, and unhealthy haze days, respectively. The concentrations of As and Cr during the hazardous haze days surpassed the PM_2.5_ Chinese class II standards (0.006 and 0.000025 μg/ m^3^).

### Airborne endotoxin measurement

**Fig. 4:**
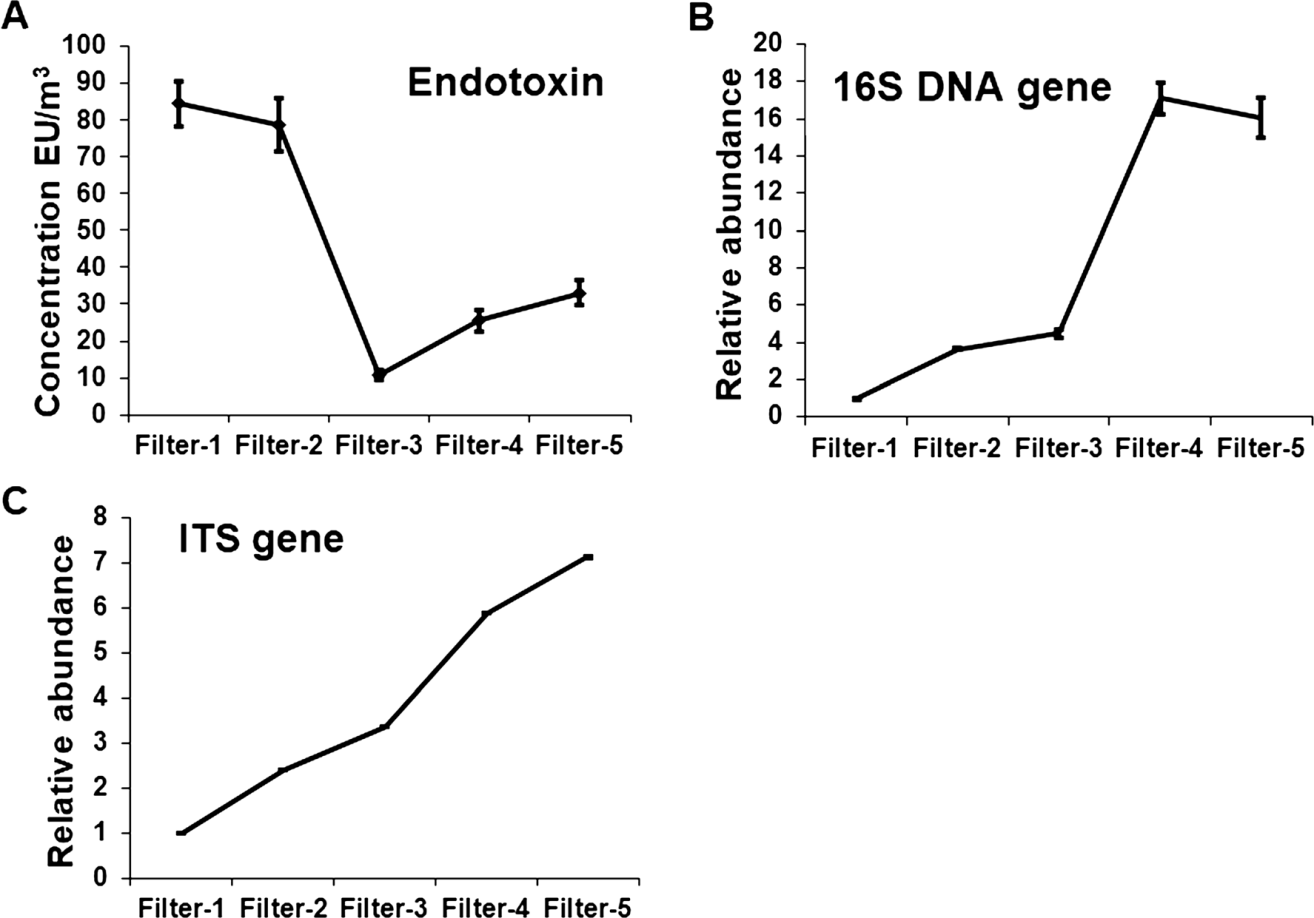
Relative concentration levels of biological components during the study period, including the endotoxin (**a**), 16S DNA gene (**b**), and ITS gene (**c**) levels

The concentration of airborne endotoxin over the study period is shown in Fig. 4a, with peak values appearing on the hazardous haze days, which had a significantly higher concentration of airborne endotoxin than the sunny days. The endotoxin concentrations on hazardous haze days, unhealthy haze days, and sunny days were 84.58, 33.07, and 10.52 EU/m^3^, respectively.

### Microbial composition analysis

**Fig. 5:**
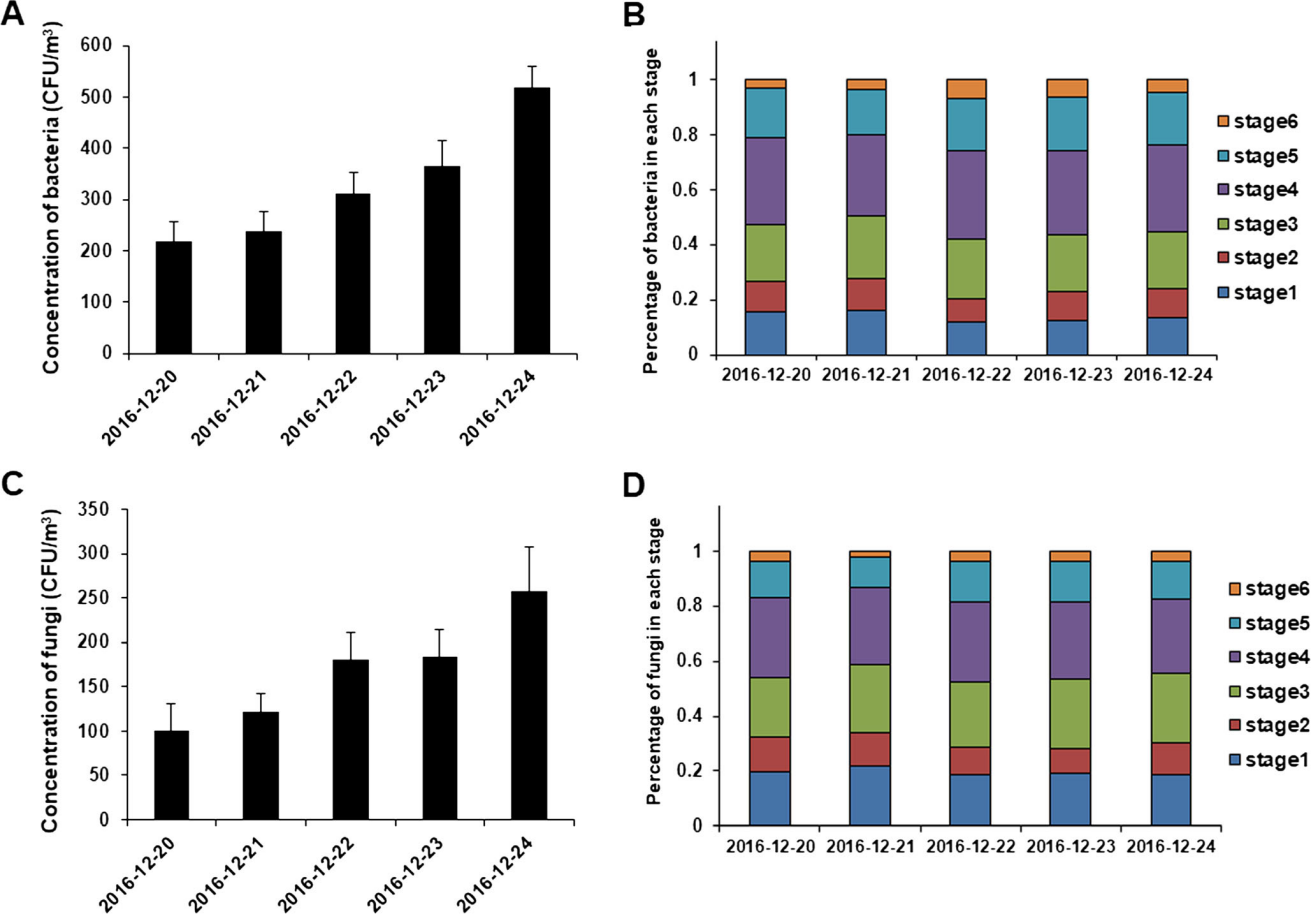
Concentration variations and average hierarchical distribution maps of bacteria (**a** and **b**) and fungi (**c** and **d**)

An ANDERSEM-6 sampler was used to identify the concentrations of culturable bacteria and fungi. Samples were collected every forenoon from December 20 to 24. Therefore, December 20 and 21 were the hazardous haze days, December 22 and 23 were the sunny days, and December 24 was the unhealthy day. Throughout the study period, the average concentrations of bacteria for the five different sampling days, in order, were 217.00 CFU/m^3^, 237.67 CFU/m^3^, 310.67 CFU/m^3^, 364.00 CFU/m^3^, and 518.00 CFU/m^3^; the average concentrations of fungi for the five different sampling days, in order, were 101.33 CFU/m^3^, 121.33 CFU/m^3^, 180 CFU/m^3^, 183.33 CFU/m^3^, and 257.33 CFU/m^3^ (Fig. 5a, c). The results indicated that the average concentration of airborne culturable bacteria and fungi differed with different AQI, following the order of unhealthy haze days> sunny days>hazardous haze days.

The results shown in the average hierarchical distribution map indicate that the bacteria and fungi had the same distribution tendency. Most of the bacteria and fungi were distributed at the fourth level (2.1–3.3 mm) and at least at the sixth level (0.65–1.1 mm). The highest distribution rates at level 4 were 32.7 and 32.3%; the lowest distribution rates at level 6 were 3.0 and 2.1%. The distribution of bacteria and fungi at levels 3 and 4 accounted for more than 50% of the total CFU (Fig. 5b, d).

To identify the relative concentration of total bacteria and fungi under different AQI, we extracted DNA from the five filters, and the relative expression levels of the 16S DNA gene and ITS gene were analyzed (Fig. 4a, b). Both of the 16S DNA gene and ITS gene concentrations were highest on unhealthy haze days and lowest on hazardous haze days, which was similar as the tendency of culturable bacteria and fungi.

**Fig. 6:**
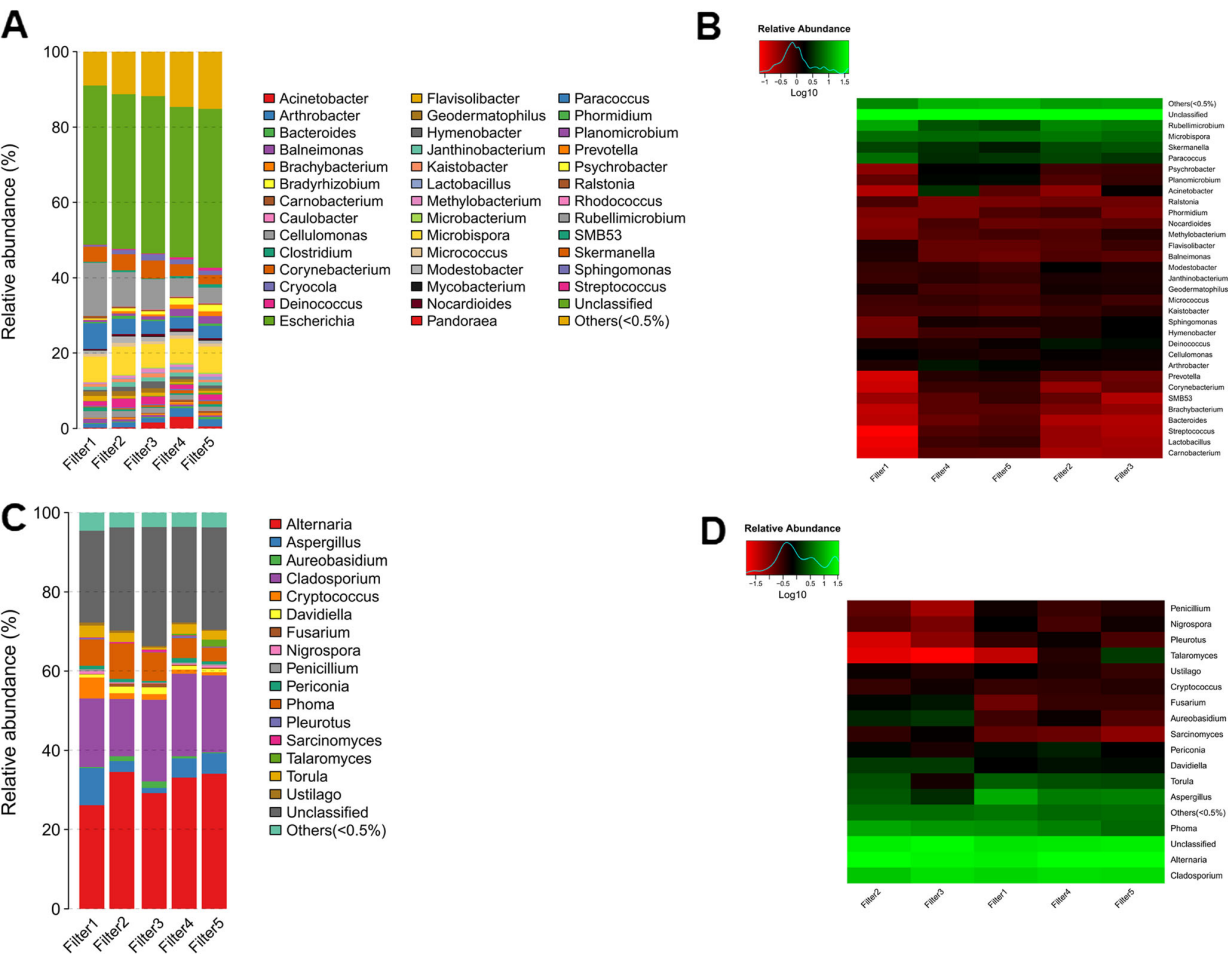
Heatmap of bacterial and fungal community structures (genera) with different abundances in the air samples. **a** and **c** represent the relative abundances of bacteria and fungi (genera), respectively. **b** and **d** represent the heatmaps of bacteria and fungi (genera), respectively. The air samples were collected during hazardous haze (Filter 1 and 2), unhealthy haze (Filter 4 and 5) and sunny days (Filter 3). The text on the right side of the heatmaps lists every bacterial or fungal genus names

In addition, the bacterial and fungal genus community structures of the five PM_2.5_ samples are shown in Fig. 6. Forty-two bacterial genera and eighteen fungal genera were detected. Except for the unclassified bacteria and fungi, the predominant bacterial genera were *Rubellimicrobium* (8.07 ± 4.00%), *Microbispora* (6.78 ± 0.48%), *Paracoccus* (4.11 ± 1.58%), and *Skermanella* (3.75 ± 0.94%). The predominant fungal genera were *Alternaria* (31.42 ± 3.63%), *Cladosporium* (18.48 ± 2.66%), *Phoma* (6.25 ± 2.12%), and *Aspergillus* (4.71 ± 3.06%). The hierarchical distribution maps and heatmaps of the bacterial and fungal class community structures with different abundances are shown in Fig. S2. The dominant bacterial classes were Alphaproteobacteria (29.85 ± 7.97%), Actinobacteria (26.14 ± 2.81%), and Betaproteobacteria (6.39 ± 1.76%). The dominant fungal classes were Dothideomycetes (76.23 ± 7.25%), Eurotiomycetes (8.07 ± 4.95%), and Sordariomycetes (4.77 ± 0.92%).

Relationship between airborne microbial composition and environmental factors (WSII, heavy metal ions, TOC and endotoxin level) was determined with redundancy analysis. However, the four environmental factors showed strong collinearity. Only TOC was left after the procedure of collinearity elimination. 30.0% of total variations in bacterial composition at the genera level and 27.1% of total variations in fungal composition at the genera level were related to TOC. The first principal component contributed to 6.31% and 2.71% of total variations in bacterial and fungal composition, respectively (Fig. S3). Some microbes, such as *Rubellimicrobium*, *Paracoccus*, *Clostridium*, *Aspergillus*, *Cryptococcus*, and *Torula*, were positively correlated with TOC, while some other microbes, such as *Sphingomonas*, *Methylobacterium*, *Acinetobacter*, *Cladosporium*, *Davidiella*, and *Aureobasidium*, were negatively correlated with TOC.

## Discussion

In the last few decades, Beijing has become an international metropolis with some common urban problems such as seasonal hazardous haze episodes induced by biomass burning. The public health problems associated with these haze episodes might be caused by many chemical or biological factors (Marlier et al. 2013). In particular, PM_2.5_ can penetrate deep into the lower respiratory tract, and the components attached to PM_2.5_ might cause toxicological effects and many other diseases associated with the respiratory and cardiovascular system (Pavagadhi et al. 2013; Yeo et al. 2014; Ho et al. 2014; Sastry 2002). In this study, the PM concentrations on the haze days were 8~10 times higher than those on the sunny days. In particular, the particles with aerodynamic diameters of 0.5–3 μm were significantly increased on hazardous haze days, which might contribute to lower respiratory diseases. In addition, most airborne microbes associated with PM with a diameter of approximately 2.1~4.7 mm might enter the nasal cavity and upper airway and then be deposited in the small bronchial tubes. Thus, people with asthma and similar diseases need to reduce outdoor exercise during haze episodes.

In the present study, NO_3_^−^ was the highest WSII species on the hazardous haze days, while SO_4_^2−^ was the highest WSII species on the sunny days. These results are consistent with those in studies by Sun and Tan (Tan et al. 2009; Sun et al. 2013) but were different from those in studies by Han and Yang (Han et al. 2015; Yang et al. 2016). SO_4_^2−^ and NO_3_^−^, which were transformed from SO_2_ and NO_x_, were used as important indicators of pollutant sources of nitrate and sulfate in the atmosphere. The increasing amount of airborne sulfate might be caused by coal consumption from the areas around Beijing (Yang et al. 2016). In addition, the concentrations of heavy metal ions on the hazardous haze days were much higher than those on the sunny days, especially the concentrations of Cr and As, which were also much higher than the PM_2.5_ Chinese class II standards; thus, their associated health hazards need to be further explored.

There are arguments about whether airborne microbial concentrations are higher on haze days than on sunny days. Some reports have found higher total microbe concentrations on hazy and foggy days than on nonhaze days (Dong et al. 2016; Haas et al. 2013). In contrast, some other reports have revealed a negative correlation between the concentrations of viable bioaerosols and PM_2.5_ (AQI) (Gao et al. 2015) or a low direct impact of air pollutants on the concentration of airborne fungal spores (Gofron et al. 2011). In our study, we found that the abundances of bacteria and fungi followed the order of unhealthy haze days>sunny days>hazardous haze days. These gaps might be explained by differences in air quality. The unhealthy haze days in this study were in the early haze period, immediately after sunny days, while the hazardous haze days followed continuous haze for 4 days. Therefore, the concentration of airborne bacteria and fungi might have been higher on the early haze days than on the sunny days and might have decreased after the haze episode had lasted for a few days. This finding is consistent with the findings of a previous study (Wei et al. 2016), which reported that biological PM (BioPM) concentrations were higher on haze days than on sunny days but decreased to the levels observed on sunny days once the haze episode had lasted for 3–5 days. During continuous haze days, the PM is usually rich with toxic and hazardous substances, which might have a negative effect on microbes. However, this finding still needed a long period monitoring for verification in future studies.

The predominant bacterial genera in our study were not similar to those of other reports (Cao et al. 2014; Wei et al. 2016). In fact, Wei et al. found that airborne bacterial structures varied significantly among studies and locations (Wei et al. 2016). Many meteorological factors, such as wind speed, temperature, relative humidity, solar radiation, and precipitation, might contribute to these differences (Bowers et al. 2011a; Bowers et al. 2011b; Bowers et al. 2012; Maki et al. 2014).

## Conclusions

In this study, we investigated the PM dynamics during a red alert air pollution event (continuous hazardous haze days) and the first few days following the event (sunny days first and then unhealthy haze days) in Beijing. The concentration of PM and its chemical components were proportional to the AQI. The percentage of particles with aerodynamic diameters of 0.5 μm–3 μm was significantly higher on haze days than on sunny days. The abundances of bacteria and fungi were higher on early haze days than on sunny days, and decreased once the haze episode had progressed for a few days. The endotoxin levels were higher on haze days than on sunny days. Most culturable bacteria and fungi were distributed in the 3rd and 4th stages (2.1–4.7 μm) of the ANDERSEM-6 sampler. Our results facilitate a better understanding of the biological and chemical composition dynamics of PM in Beijing and provide useful data for health impact evaluations during haze episodes.

## Electronic supplementary material

Figure S1 Paired pictures of hazardous haze days and sunny days (DOCX 227 kb)

Figure S2 Heatmap of bacterial and fungal community structures (classes) with different abundances in the air samples. (A) and (C) represent the relative abundances of bacteria and fungi (classes), respectively. (B) and (D) represent the heatmaps of bacteria and fungi (classes), respectively. The air samples were collected during hazardous haze (Filter 1 and 2), unhealthy haze (Filter 4 and 5) and sunny days (Filter 3). The text on the right side of the heatmaps lists every bacterial or fungal class name. (DOCX 306 kb)

Figure S3 Plot of redundancy analysis (RDA) of the airborne bacterial composition (A) and fungal composition (B) at the genus level relative to total organic carbon (TOC). Only taxa with an average relative abundance ≥1% in at least one sample were involved. Constrained explanatory variable (TOC) was indicated by blue arrow. (PNG 199 kb)

High resolution image (TIF 421 kb)

Table S1 Levels of the chemical compositions measured in the five air samples during the sampling campaign (DOCX 17 kb)
