# Supplementary figures and images for: Biological and chemical compositions of atmospheric particulate matter during hazardous haze days in Beijing
Source: Environ Sci Pollut Res Int. 2018 Oct 12;25(34):34540–9. doi: 10.1007/s11356-018-3355-6 (PMC6245000; doi:10.1007/s11356-018-3355-6)

**Figure S1. Paired pictures of hazardous haze days and sunny days**


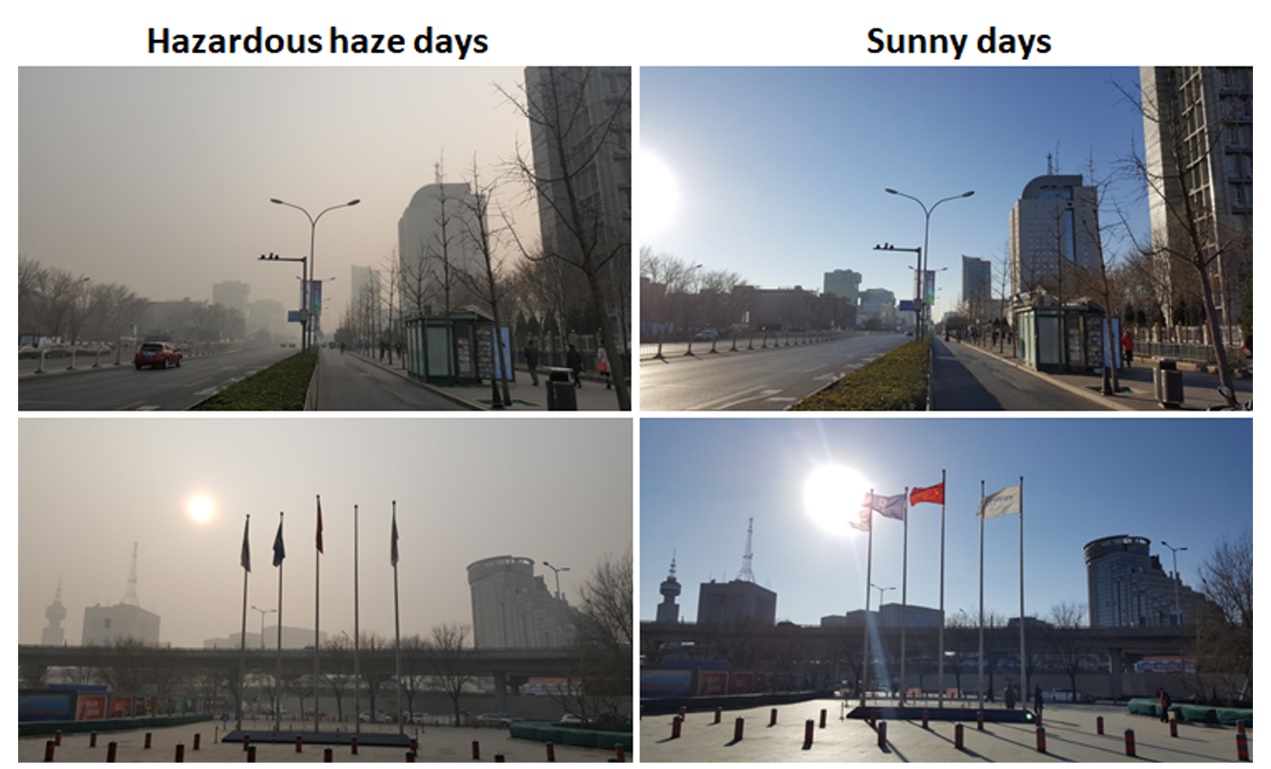

Supplement: Supplementary file 1 — Paired pictures of hazardous haze days and sunny days (DOCX 227 kb) [file 11356_2018_3355_MOESM1_ESM.docx]

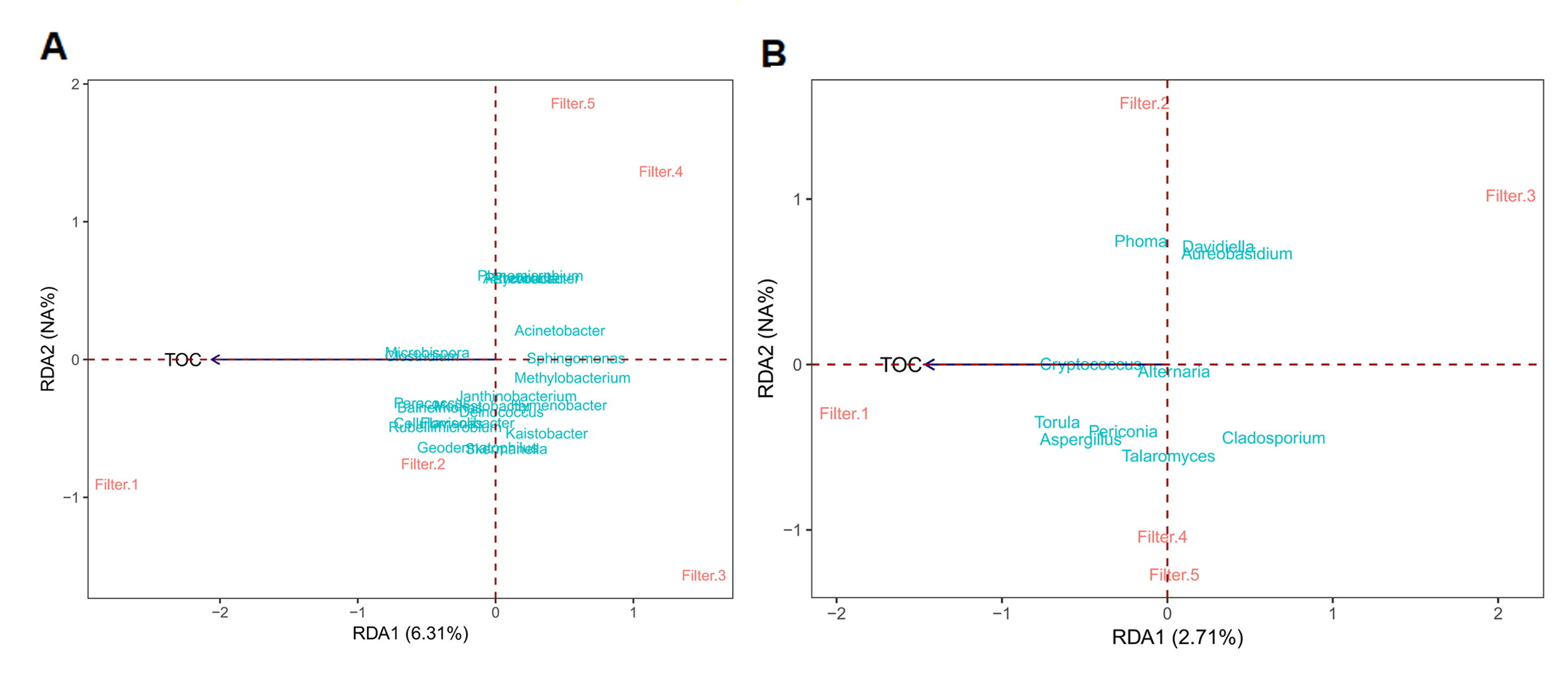

Supplement: Supplementary file 3 — Plot of redundancy analysis (RDA) of the airborne bacterial composition (A) and fungal composition (B) at the genus level relative to total organic carbon (TOC). Only taxa with an average relative abundance ≥1% in at least one sample were involved. Constrained explanatory variable (TOC) was indicated by blue arrow. (PNG 199 kb) [file 11356_2018_3355_Fig7_ESM.png]

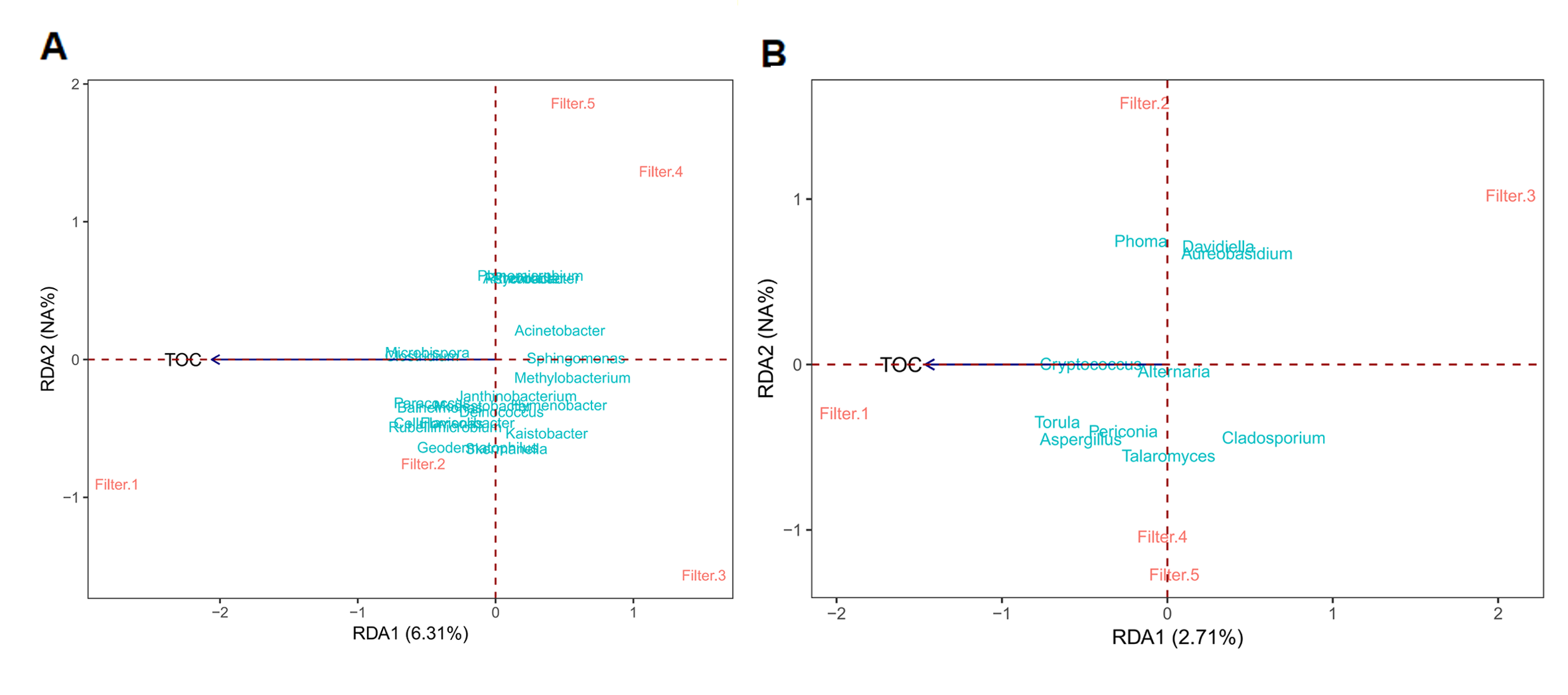

Supplement: Supplementary file 4 — High resolution image (TIF 421 kb) [file 11356_2018_3355_MOESM3_ESM.tif]
