# Supplementary material for: Biological and chemical compositions of atmospheric particulate matter during hazardous haze days in Beijing
Source: Environ Sci Pollut Res Int. 2018 Oct 12;25(34):34540–9. doi: 10.1007/s11356-018-3355-6 (PMC6245000; doi:10.1007/s11356-018-3355-6)

**Figure S2. Heatmap of bacterial and fungal classes community structures with different abundances in the air samples. (A)** and **(C)** represent the relative abundances of bacteria and fungi, respectively. **(B)** and **(D)** represent the heatmap of bacteria and fungi, respectively. The air samples were collected during hazardous haze (Filter 1 and 2), unhealthy haze (Filter 4 and 5) and sunny days (Filter 3). The text on the right side of these heatmaps is the classes names for every bacteria or fungi.
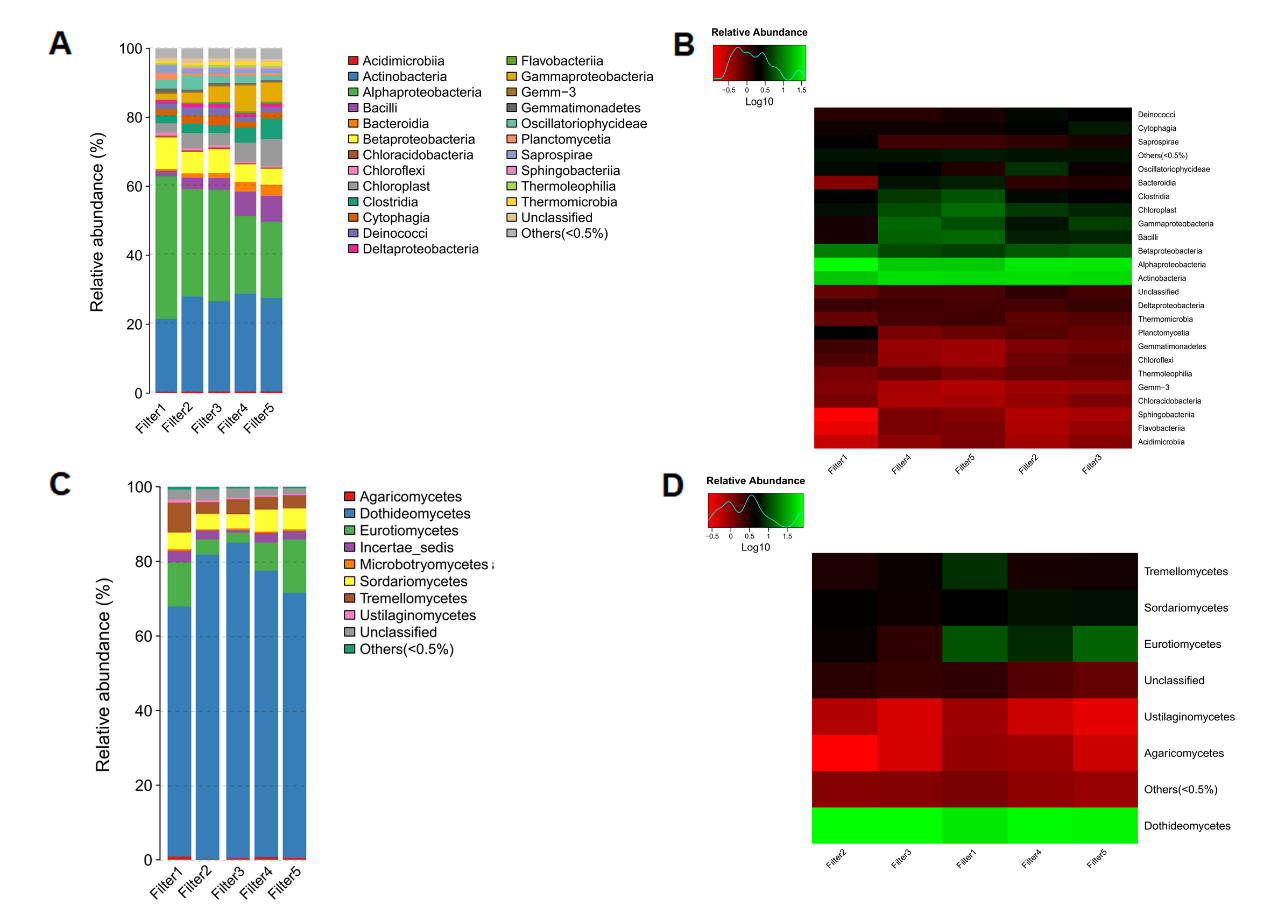

Supplement: Supplementary file 2 — Heatmap of bacterial and fungal community structures (classes) with different abundances in the air samples. (A) and (C) represent the relative abundances of bacteria and fungi (classes), respectively. (B) and (D) represent the heatmaps of bacteria and fungi (classes), respectively. The air samples were collected during hazardous haze (Filter 1 and 2), unhealthy haze (Filter 4 and 5) and sunny days (Filter 3). The text on the right side of the heatmaps lists every bacterial or fungal class name. (DOCX 306 kb) [file 11356_2018_3355_MOESM2_ESM.docx]
