# Supplementary material for: Biological and chemical compositions of atmospheric particulate matter during hazardous haze days in Beijing
Source: Environ Sci Pollut Res Int. 2018 Oct 12;25(34):34540–9. doi: 10.1007/s11356-018-3355-6 (PMC6245000; doi:10.1007/s11356-018-3355-6)
Supplement: Supplementary file 5 — Levels of the chemical compositions measured in the five air samples during the sampling campaign (DOCX 17 kb) [file 11356_2018_3355_MOESM4_ESM.docx]

**Table S1. Levels of the chemical compositions measured in the five air samples during the sampling campaign**

| **Category** | **ion** | **Concentration (μg/m^3^)** | | | | |
| --- | --- | --- | --- | --- | --- | --- |
|  |  | **Filter-1** | **Filter-2** | **Filter-3** | **Filter-4** | **Filter-5** |
| **Carbon** | **TOC** | **26.91** | **22.66** | **5.23** | **9.26** | **14.41** |
| **Water-soluble inorganic ions** | **Ca^2+^** | **8.93** | **4.64** | **2.46** | **2.80** | **5.92** |
|  | **Mg^2+^** | **1.39** | **0.76** | **0.23** | **0.32** | **0.68** |
|  | **K^+^** | **2.08** | **1.74** | **0.21** | **0.49** | **1.14** |
|  | **NH_4_^+^** | **22.35** | **35.71** | **1.26** | **4.66** | **10.24** |
|  | **Na^+^** | **17.65** | **2.79** | **0.33** | **0.61** | **1.18** |
|  | **SO_4_^2-^** | **32.01** | **33.30** | **2.96** | **4.91** | **10.60** |
|  | **Cl^-^** | **8.76** | **6.48** | **0.47** | **1.92** | **4.64** |
|  | **NO_3_^-^** | **43.74** | **47.37** | **1.63** | **6.70** | **13.39** |
|  | **Zn^2+^** | **0.25** | **0.25** | **0.032** | **0.052** | **0.11** |
|  | **Cu^2+^** | **0.0025** | **0.0016** | **0.00013** | **0.00028** | **0.0010** |
|  | **Fe^3+^** | **0.55** | **0.52** | **0.10** | **0.14** | **0.41** |
|  | **Al^3+^** | **0.69** | **0.32** | **0.097** | **0.11** | **0.31** |
|  | **Ni^2+^** | **0.00026** | **0.00017** | **0.00012** | **0.00010** | **8.99E-05** |
| **Heavy metal ions** | **Hg** | **0.0026** | **0.0025** | **0.00013** | **0.00013** | **0.00013** |
|  | **Pb** | **0.019** | **0.014** | **0.00073** | **0.0030** | **0.0059** |
|  | **Cd** | **0.00057** | **0.00071** | **3.91E-05** | **6.81E-05** | **0.00019** |
|  | **As** | **0.0061** | **0.0077** | **0.00088** | **0.0036** | **0.0057** |
|  | **Cr** | **0.00026** | **0.00017** | **<0.00014** | **<0.00014** | **<0.00014** |
